# Intra Nasal *In situ* Gelling System of Lamotrigine Using Ion Activated Mucoadhesive Polymer

**DOI:** 10.2174/1874104501711010222

**Published:** 2017-12-29

**Authors:** Asha Paul, K.M .Fathima, Sreeja C. Nair

**Affiliations:** Department of Pharmaceutics, Amrita School of Pharmacy, Amrita Vishwa Vidyapeetham, Amrita University, AIMS Health Science Campus, Kochi, India

**Keywords:** Intranasal formulation, Mucoadhesion, *Insitu* gel, Immediate release, Acute epileptic condition, *Ex vivo* permeation

## Abstract

**Background:**

A novel drug delivery system for treating acute epileptic condition.

**Objective:**

To develop an intranasal mucoadhesive formulation of Lamotrigine (LTG) loaded *insitu* gel, for the treatment of epilepsy to avoid possible side effects and first pass metabolism associated with conventional treatment.

**Methods:**

Lamotrigine was loaded into different polymeric solutions of gellan and xanthan gum.

**Results:**

All formulations subjected to various evaluation studies were within their acceptable limits. The pH of formulation ranges between 5.8 ±.001 to 6.8 ±.005 indicating that no mucosal irritation is expected as pH was in acceptable range. *Invitro* drug release from the mucoadhesive *insitu* gel formulations showed immediate drug release pattern with a maximum drug release of 97.02 ±0.54% for optimized G5 formulation within 20min. *Exvivo* permeation studies of optimized formulation G5 and control formulation was estimated. *Exvivo* permeation studies of G5 *insitu* formulation done for a period of 12 h resulted in slow, sustained release and greater permeability significance(P <0.05) through nasal mucosa when compared to control. Histopathological studies showed that G5 formulation was safer for nasal administration without any irritation. The stability studies indicated that gels were stable over 45 days in refrigerated condition (4±2ºC).

**Conclusion:**

The intranasal *insitu* gelling system is a promising novel drug delivery system for an antiepileptic drug lamotrigine which could enhance nasal residence time with increased viscosity and mucoadhesive character and provided better release profile of drug for treating acute epileptic conditions.

## 1 INTRODUCTION

Epilepsy is a neurological disorder of brain characterized by sudden recurrent episodes of sensory disturbances with brief loss of consciousness and awareness, which ultimately lead to seizures. Epileptic seizures are undetectable and can vary from short to long periods of vigorous shaking, to loss of body functions and motor functions and to mood swings.

In epilepsy, seizures tend to recur and have no immediate underlying cause [1]. Epileptic seizures are the result of excessive and abnormal nerve cell activity in the cortex of the brain. Pathophysiology of epilepsy is partially understood [2]. Some occur as a result of tumour of brain, brain injury and brain defect stroke. About 50 million people have epilepsy worldwide. Seizures can be controlled with medication in about 70% cases otherwise surgery, dietary changes and neurostimulation should be considered. AEDs (Antiepileptic drugs) are the medications used in the treatment of epileptic seizure, some of them act by targeting the voltage gated Na^+^ (sodium) and Ca^+^ (calcium) channel while some act in stimulating GABA system. Selection of AED for a patient depends upon the affected individual’s condition and side effects of drug. This article mainly helps in evaluating and developing an *insitu* gel loaded with lamotrigine. The reason for selecting intranasal *insitu* drug delivery is because of its large surface area for drug absorption, as it avoids first pass metabolism and it help in delivering drug to CNS thereby by passing BBB, there is rapid onset of pharmacological action and higher bioavailability of lipophilic drugs like lamotrigine. *Insitu* delivery system favours the ease and convenience of administration as drops allowing accurate dosing. Mucoadhesive system aims in targeting and localization of the dosage forms and provide an intimate contact between dosage form and absorptive mucosa resulting in high drug flux at the absorbing tissue. It’s a novel drug delivery system since lamotrigine was only available as tablet (chewable, disintegrating, extended release) and IV formulation. Intranasal *insitu* gel is developed for the purpose of immediate release of lamotrigine for treating acute epileptic condition, which is very essential in treating an emergency condition [3-24].

## 2 METHODOLOGY

### 2.1 Materials

Lamotrigine was provided as a gift sample by Auro Bindo Pharma private limited, Hyderabad. Gellan gum was obtained from Marine Hydrocolloids, Kochi. Dialysis membrane (12000-14000 mol. Wt. Cut off) was procured from Sigma Aldrich.

### 2.2 Preformulation Studies

Preformulation investigations are intended to convey all essential data, especially physico-chemical, physico-mechanical and biopharmaceutical properties of drug substances and its excipients as well as its compatibility.

#### 2.2.1 Identification of Drug

##### 2.2.1.1 Fourier transform infrared (FTIR) spectroscopy [25-27]

FTIR spectrum of the obtained pure drug of Lamotrigine was compared with the FTIR of the standard drug spectrum (from monograph, IP). FTIR spectrum was obtained by using FTIR spectrophotometer by KBr (potassium bromide) pellet method.

##### 2.2.1.2 Solubility Studies [28, 29]

Solubility studies of drug lamotrigine) was determined in different solvents such as water, ethanol, methanol, 1-propanol, acetone and 1-butanol and in phosphate buffer (PBS) pH 6.6 to find the solvent in which lamotrigine is completely soluble, so as to give a better absorption curve of lamotrigine in UV spectrophotometer.

##### 2.2.1.3 Melting Point [30, 31]

The purity of the sample is indicated by its melting point. The presence of relative slight amount of impurity will decrease the melting point. Open capillary method was used to determine the melting point of sample.

##### 2.2.1.4 Partition Coefficient [32, 33]

Partition coefficient of Lamotrigine in n-octanol water was determined. Equal volumes of water and n-Octanol were taken in a separating funnel, and known amount of lamotrigine was added in it. For 2h at constant temperature the funnel was vigoursly shaken at regular intervals. Then the amount of drug in aqueous layer was determined by UV spectroscopy (Ultra Violet Spectroscopy) at 309nm (Nano meter). The n-octanol partition coefficient of the drug was obtained using the following equation:

Partition coefficient = Concentration of drug in organic layer/ Concentration of drug in aqueous phase.

##### 2.2.1.5. Lamda Max (ʎ _max_) of Lamotrigine in Methanol [34]

Absorption maxima of lamotrigine was determined in solvent methanol. Standard stock solution is prepared by dissolving 10mg (Milligram) of lamotrigine in 30ml (Millilitre) methanol by sonication for 10 minutes and volume was made up to 100ml mark using methanol. The concentration of standard stock solution is 100µg/ml (Microgram per millilitre). This was scanned from 400-200nm by using UV spectrophotometer.

##### 2.2.1.6 Lamda Max of Lamotrigine in PBS pH 6.6

Absorption maxima of lamotrigine was determined in solvent PBS pH 6.6. Standard stock solution was prepared by dissolving 10mg of lamotrigine in 30ml PBS pH 6.6 by sonication for 10 minutes and volume was made up to 100ml mark using PBS pH 6.6. The concentration of standard stock solution was 100µg/ml. This was scanned from 400-200nm by using UV spectrophotometer.

##### 2.2.1.7 Compatibility Studies of Drug Lamotrigine with the Excipients [35, 36]

It is very important to carefully select the excipients to develop an effective, stable and optimised dosage form to facilitate easy administration, promote the better release and bioavailability of drug and to protect it from degradation. The compatibility study of drug lamotrigine and excipients is very important. The drug excipient compatibility study was performed by preparing a homogenous physical mixture of drug and all possible excipients to be used in formulation in the ratio 1:1. FTIR spectra of drug and excipients were obtained to ascertain the compatibility between lamotrigine and selected polymers using FT infrared spectrophotometer by KBr pellet method.

##### 2.2.1.8 X-ray Diffraction Studies (XRD) [37-39]

XRD studies are the most powerful and established technique for material structural analysis, capable of providing information about the structure of a material at its atomic level. X-Ray diffraction studies pattern was recorded for a specified quantity of pure drug and optimised formulation (G5) on X-ray diffractometer (Model –Bruker AXS D8 advance, Configuration – Vertical theta /2 theta geometry, Angle range - 360˚, X Ray source - Cu, Wavelength - 1.5406 ˚A, Detector – Si (Li) PSD, Temperature range -170˚C - 450˚C). The XRD pattern of *insitu* gel formulation was compared with that of the pure drug.

#### 2.2.2 Analytical Methods

Calibration curve was done using methanol and PBS pH 6.6.

##### 2.2.2.1 Calibration Curve of Lamotrigine in Methanol [40]

- Preparation of standard stock solution
  10mg of lamotrigine in 30ml of methanol was sonicated for 10 minutes and the volume was made up to 100ml mark using methanol. The drug was dissolved and diluted with methanol to get concentration of 100µg/ml.
- Preparation of standard graph
  From this stock solution(100µg(microgram)) different aliquots 0.5ml,1ml,1.5ml,2ml and 2.5ml were drawn and diluted to 10ml with methanol separately to prepare a series of concentration from range 5-30µglml. The standard stock solution (100µglml) was scanned in the range of 400-200nm against methanol as blank. Standard graph was obtained by plotting absorbance against concentration (µg/ml).

##### 2.2.2.2 Calibration Curve of Lamotrigine in PBS pH 6.6

- Preparation of PBS pH 6.6 solution 2.712g (gram) of potassium dihydogen ortho phosphate was dissolved in 100ml distilled water and 0.8g of sodium hydroxide (NaOH) was dissolved in 100ml distilled water separately. From the prepared solutions, 25ml of potassium dihydogen ortho phosphate and 8.2ml of NaOH were pipetted into 100ml standard flask and volume was made up to 100ml mark using distilled water.
- Preparation of standard stock solution
  10mg of lamotrigine in 30ml of phosphate buffer was sonicated for 10 minutes and the volume was made up to 100ml mark using PBS. Using PBS the drug was dissolved and diluted to get a concentration of 100µglml.
- Preparation of standard graph

From this stock solution (100µg) different aliquots were drawn and diluted to 10ml with PBS separately to prepare a series of concentration from range 5-30µglml. The standard stock solution (100µglml) was scanned in the range of 400-200nm against PBS as blank. Standard graph was obtained by plotting absorbance against concentration (µg/ml).

### 2.3 Formulation of Intra Nasal *In situ* Gel of Lamotrigine [41-49]

Polymeric solution of gellan gum and xanthan gum was prepared using deionised water and mixed with mechanical stirrer for 2 min. Preservatives, such as ethyl paraben, mannitol and Polyethylene glycol (PEG), were added into polymeric solutions, and then kept in a water bath for few minutes. Then, it was cooled to room temperature. Drug was then mixed with small amount of methanol and then sonicated. Drug solution was added to beaker containing polymeric mixture which was then continuously stirred until the entire drug was dissolved. The prepared formulation was transferred to a clean glass container which was made up to 10ml volume with distilled water and stored in a cool place. Table **1** gives the composition of *insitu* gel of lamotrigine.

### 2.4 Characterisation of Intranasal *In situ* Gel

#### 2.4.1 Physiochemical Properties

##### 2.4.1.1 Homogeneity Studies

All the prepared formulations were inspected for homogeneity studies by visually observing the appearance and the presence of lumps in the formulation.

##### 2.4.1.2 Gelling time [50, 51]

The system exists in sol form before administration and after administration it turns into gel form. Gelling time is the time taken for the transformation of sol to gel form. Time for gelation is recorded by placing 2ml of prepared solution in a test tube and adding small amount of simulated nasal fluid, maintaining the temperature at 37ºC and visually observing the gel formation, and onset of time for gelation is recorded as gelling time in seconds.

##### 2.4.1.3 Syringeability

Syringeablity test was performed by transferring freshly prepared formulations into an identical 5 ml plastic syringe placed with 20 gauge needle to a constant volume (1 ml). The solutions which were easily passed from syringe weretermed as pass and difficult to pass were termed as fail.

##### 2.4.1.4 pH Study [52-55]

One ml of the prepared *insitu* gels was diluted with distilled water. A Digital pH meter (Shambhavi Impex, India) which was previously calibrated was used for determination of pH of the resulting solution.

##### 2.4.1.5 Spreadability [56]

Spreadability was determined by placing excess of sample *insitu* gel between two glass slides and for 5 min under tension by placing 1000g weight, the slides were compressed to uniform thickness. Spreadability is the time in seconds for two slides to slip off from *insitu* gel which was placed in between the slides. The slides are under the influence of certain load. The spreadability is expressed in g cm sec^-1^ (gram centimetre/seconds).

##### 2.4.1.6 Gel Strength [57, 58]

Gel strength was determined by using texture analyser (Model – TA.XT2 Plus, Size- 650mm tall, 540mm deep, 280mm wide, Speed – 0.01- 40mm/sec, Maximum aperture -370mm,Sample testing area- 247mm x 228mm, load cell capacity - 3g- 10kg, Operating temperature- 0-40˚C). The experiment was done by placing *insitu* gel in standard beaker below probe. In this, an analytical probe was depressed into the sample. The texture analyser was set to gelling strength test mode or compression mode with test speed of 1.0mm/sec, an acquisition rate of 50 points per sec and trigger force of 3-5g were selected. An aluminium probe of 7.6cm diameter was used for all the prepared formulations. The study was carried out at room temperature and the force required to penetrate the gel was measured as gel strength in grams.

##### 2.4.1.7 Viscosity Studies [59, 60]

Viscosity of *insitu* gel system was determined using Brook field viscometer DV-1 prime and Pro couple with S-94 spindle. Temperature of 37±0.5ºC was maintained and the spindle was lowered perpendicularly into both *insitu* sol and gel formulations which were placed in a beaker. The viscosity of each formulation was determined by applying 100rpm speed.

##### 2.4.1.8 Rheological Studies [61-65]

The measurement of viscosity of prepared *insitu* gel was done with Brookfield viscometer. The *insitu* formulations were rotated for 2 minutes at different speeds for selected spindle. At each speed the corresponding dial reading was noted. The viscosity of different *insitu* gel formulations was measured at different speeds at room temperature. A typical run should be carried out by changing the speed from 10-100 rpm (revolutions per minute).

##### 2.4.1.9 Drug Content Estimation [66, 67]

Suitable dilutions of drug in PBS was made by dissolving 1ml of formulation in 10ml PBS of pH 6.6 which was taken in 10ml volumetric flask. The drug content was estimated on UV visible spectrophotometer at 309nm using PBS pH 6.6 as blank.

### 2.5 *In vitro* Mucoadhesion Studies

#### 2.5.1 *In vitro* Mucoadhesion Time [68, 69]

*Invitro* mucoadhesion studies were performed to evaluate the mucoadhesion properties of *insitu* gel loaded with lamotrigine. The time required for the detachment of *insitu* gel spread on the nasal mucosa was determined. The tissues were kept in PBS and stored in freezer and the same was kept at room temperature before doing the test. Sections of nasal mucosa were fixed onto a glass beaker with mucosal surface facing outwards. A small quantity of nasal *insitu* formulation was placed on the mucosal surface previously treated with simulated nasal fluid. Mucosa fixed on to the glass beaker was then placed in the beaker containing 100ml of PBS and was magnetically stirred at 10 rpm. The time required for erosion of *insitu* gel was noted visually and considered as the *invitro* mucoadhesion time.

#### 2.5.2 *In vitro* Mucoadhesive Strength [70-82]

The force used to detach the *insitu* gel placed in between the two nasal mucosae was determined with the help of a specialised chemical balance. The nasal mucosa mainly the bovine mucosa is used for the study. On one side of the balance the nasal mucosa is tied on a clear glass surface with help of a rubber band and another mucosa was placed in such a way that the mucosa is in inverted position to that of the first, so that both the mucosal surfaces face each other. A small quantity of the prepared *insitu* gel about 50mg is placed in between the two mucosae and allowed to remain in contact with each other for few minutes. For each measurement using different formulation, different mucosa was used. On the other side of the pan, weight was increased until the two mucosae were detached from each other. Mucoadhesive strength is expressed as force or stress detachment per cm square of area of mucosa used.

It is given by the equation dynes/cm^2^ =mg/A;

Where m=Weight in grams required for the detachment of two mucosae.

g =acceleration due to gravity.

A=Area of mucosa exposed.

### 2.6 *In vitro* Release Studies [83-85]

*Invitro* drug release study was performed by static dissolution method. *In vitro* drug release was carried out by placing the prepared *insitu* gel formulations in a beaker containing the 50ml of PBS pH6.6. The temperature was maintained at 37±0.5ºC throughout the study. Gel form was initially prepared by treating the *in situ* formulation with simulated nasal fluid (composition include sodium chloride -0.745gm, potassium chloride -0.12gm, calcium chloride dehydrated- 0.005mg distilled water -100ml)which was then placed in the beaker containing PBS. Then beaker was placed on magnetic stirrer and stirred for adequate period of time. Aliquots of samples were withdrawn at regular intervals and the dissolution medium was replaced with fresh PBS. The samples withdrawn were suitably diluted with PBS pH6.6 and the amount of drug released was analysed spectrophotometrically at 309nm against PBS pH 6.6 as blank. After the *in vitro* release studies, the formulation with maximum release was found and the kinetic studies were done by subjecting the release data of the optimised formulation to different kinetic models and calculated the corresponding regression coefficient values (R^2^).

### 2.7 *Ex vivo* Permeation Study [86-93]

Franz diffusion cell was used for doing the *exvivo* permeation study. The diffusion cell has receptor compartment of 15ml capacity which was filled with PBS of pH6.6. Bovine nasal mucosal tissue used in *ex vivo* permeation was obtained from local slaughter house and cut in suitable shape. Bovine mucosa was selected since it resembles human nasal mucosa. Mucosal tissue was properly placed over the receptor compartment. From 20 mg containing 10ml of optimised formulation 1ml of *in situ* gel formulation was placed in the donor compartment. Aliquots of samples were withdrawn from the receptor compartment at regulator intervals and fresh samples were replaced into receptor compartments. Samples were suitably diluted and drug content was analysed spectrophotometrically at 309nm. The permeation study was done for optimised G5 *insitu* formulation and control containing drug in PBS pH 6.6 and comparison was done among them for steady state flux value for a maximum period of 12h. Statistical analysis of the data was carried out by performing Students T Test.

### 2.8 Histopathological Study [94-96]

The pre-treated mucosa after in vitro permeation was subjected to histopathological examinations for knowing any changes after accepted procedure. Nasal mucosa treated with control and optimised G5formulation were subjected to histopathological examination using a light microscope. 10% buffered formalin (pH 7.0) used to fix tissue was routinely processed and embedded in paraffin. Sections (5 µm) were cut on glass slides and stained with hematoxylin and eosin.

### 2.9 Scanning Electron Microscopy (SEM) [97]

The optimised insitu G5 formulation was suitably diluted and examined with SEM.

### 2.10 Transmission Electron Microscopy (TEM) [98]

The optimised *insitu* G5 formulation was subjected to TEM, in which a beam of electrons was transmitted through the sample to form an image.

### 2.11 Stability Studies [99, 100]

The optimised *insitu* gel formulation (G5) underwent stability evaluation for 45 days in refrigerated (4±2ºC), room temperature (30±2ºC) and oven (40±2ºC) conditions and analysed for drug content and gel strength at regular intervals. Mean values were recorded.

## 3 RESULTS AND DISCUSSION

### 3.1 Preformulation Studies

#### 3.1.1 FTIR Spectroscopy

FTIR of pure drug lamotrigine in Fig. (**1**) was found to be in accordance with monograph (IP).

#### 3.1.2 Solubility Studies

The pure drug is partially soluble in water and ethanol and completely soluble in methanol, acetone, PBS pH 6.6.

#### 3.1.3 Melting Point

The melting point of the drug was found to be 225-235 °C and it was in accordance with that of the reference.

#### 3.1.4 Partition Coefficient

Partition coefficient of the drug was found to be 1.1 indicating that the drug is practically insoluble in water and has high lipophlilicity.

#### 3.1.5 Lambda max of Lamotrigine in Methanol

The absorption maxima of lamotrigine in methanol was found to be 309nm as shown in Fig. (**2a**), which was in accordance with the official standard.

#### 3.1.6 Lambda Max of Lamotrigine in PBS pH6.6

**Fig. (2):**
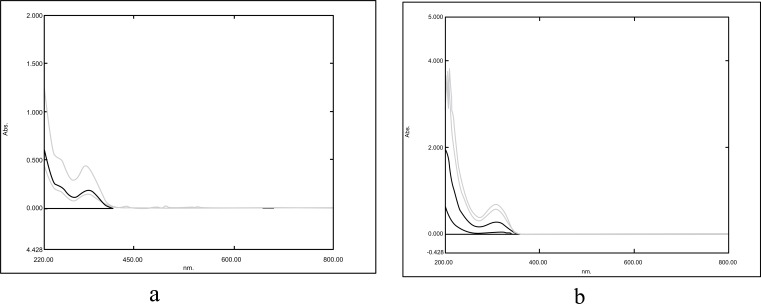
a) Absorption maxima of lamotrigine in methanol; and b) Absorption maxima of lamotrigine in PBS pH 6.6.

The absorption maxima of lamotrigine in PBS pH 6.6 was found to be 309 nm (Fig. **2b**).

#### 3.1.7 Compatibility Studies of the Drug Lamotrigine with the Excipients

**Fig. (3):**
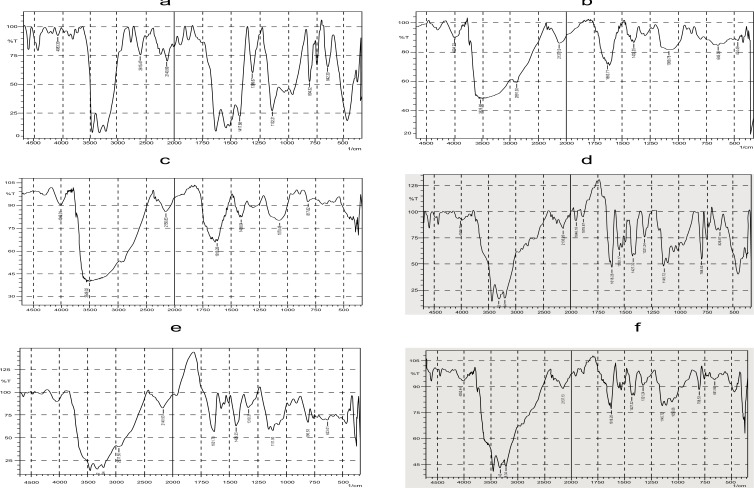
FTIR spectra of a) lamotrigine; b) gellan gum; c) xanthan gum; d) drug with gellan gum; e) drug with xanthan gum; and f) *in situ* gel formulation.

FTIR spectra of lamotrigine, gellan gum, xanthan gum, drug with gellan gum, drug with xanthan gum, *insitu* gel formulation are shown in (Fig. **3**).

- FTIR spectra of Lamotrigine shown in Fig. (**3a**) showed characteristic absorbance at 3446 cm^-1^ specifying NH stretching of amino group, 3211cm^-1^ shows aromaticity (aromatic CH stretching), 1627 cm^-1^, 1417cm indicates C=C ring stretching, and 843.02 cm^-1^ shows C-Cl stretching of halides .
- FTIR spectra of gellan gum shown in Fig. (**3b**) showed a broad strong peak at 3525 cm^-1^ indicating the presence of OH group, and 2891cm^-1^ shows aromatic CH stretching.
- FTIR spectra of Xanthan gum shown in Fig. (**3c**) also showed a broad peak at 3549 cm^-1^ which also relieved the presence of OH group.
- FTIR spectra of *insitu* gel formulation exhibited a sharp peak at 3315 cm^-1^ which unveiled the existence of amino group (NH stretching of NH group), 325.34 cm^-1^ indicates aromatic CH stretching, 1627 cm^-1^, 1427cm^-1^ shows C=C ring stretching, 798-659 cm^-1^ displays C -Cl stretching of Halides, and a strong absorbance peak at 3445 reveals the presence of OH group.
- Absence of extra peaks indicates good compatibility between drug and excipients.

#### 3.1.8 XRD Studies

The XRD pattern of both *insitu* formulation and lamotrigine is shown in Figs. (**4a** and **4b**), respectively. The characteristic XRD patterns demonstrate the crystalline nature of lamotrigine. XRD pattern of lamotrigine has sharp and intense peak appearing at 12.1, 13.2, 24.9, 27.5 2 theta values. While comparing the XRD pattern of drug with the *insitu* formulation, the latter showed a decrease in the intensity and number of peak. This clearly indicates the degree of amorphous nature of formulation. The crystalline nature of lamotrigine was reduced in the *insitu* formulation.

### 3.2 Analytical Methods

#### 3.2.1 Calibration Curve of Lamotrigine in Methanol

**Fig. (5):**
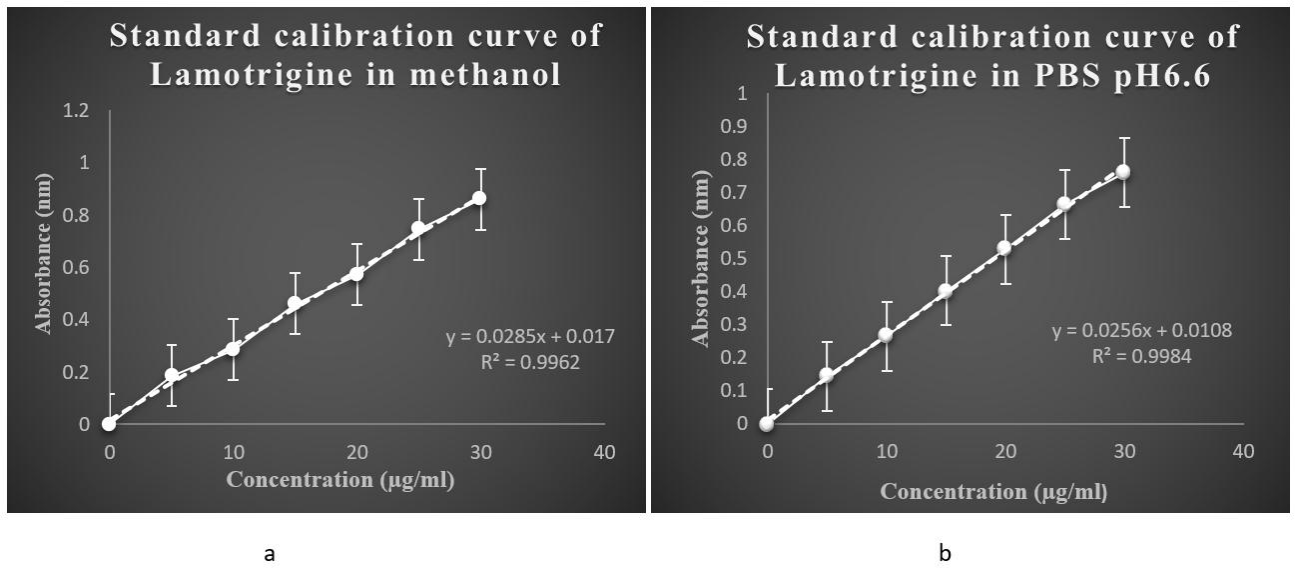
a) Standard calibration curve of lamotrigine in methanol, b)Standard calibration curve of lamotrigine in phosphate buffer pH 6.6.

The calibration curve of lamotrigine in methanol was found to be linear in concentration range of 5-30 µg/ml. The calibration curves are shown in (Fig. **5a**).

#### 3.2.2 Calibration Curve of Lamotrigine in PBS pH 6.6

The calibration curve of lamotrigine in phosphate buffer pH6.6 was found to be linear in concentration range of 5-30µg/ml. The calibration curves are shown in (Fig. **5b**).

### 3.3 Characterisation

#### 3.3.1 Homogeneity Studies

All the prepared *in situ* gels showed noble homogeneity with the absence of lumps indicating uniform drug distribution of the formulation.

#### 3.3.2 Gelling Time

**Fig. (6):**
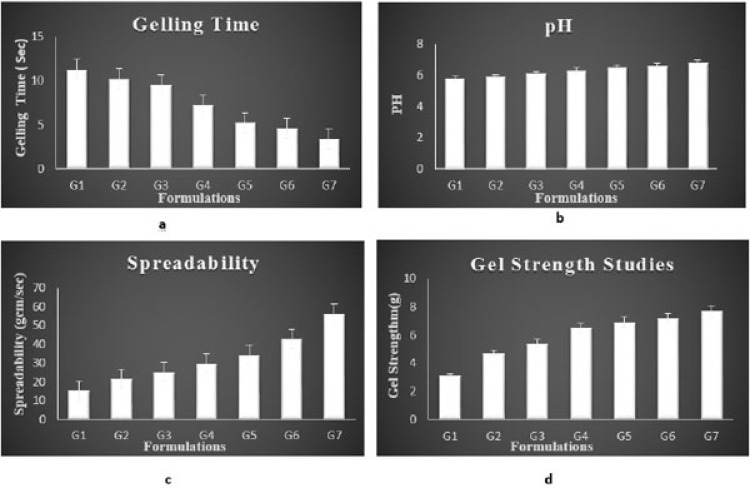
a) Showing gelling time; b) pH; c) Spreadability; d) Gel strength of different formulations of *insitu* gel of lamotrigine.

The gelling time of the formulation ranged between 3.4 ± 0.21 sec and 11.3 ± 0.22 sec. The formulations G5 showed an adequate gelling time. Gelling time of all formulations is mentioned in Table **2** and Fig. **6a**.

#### 3.3.3 Syringeablity

All the formulations from G1- G5 passed syringeability test and showed good syringeability except G6 and G7as shown in Table (**2**). Syringeablity test was conducted for ensuring that the prepared formulation was having proper solution flow nature so that the formulation can be used to deliver the *insitu* sol to the target site. The syringeability depends on 3 parameters mainly viscosity of the system, flow rate, and needle characteristics. Syringeablity mainly depends on the concentration of the polymer and viscosity. Since the former ones especially G5 and G4 were having lesser polymer concentration which exhibited good Syringeablity. As the polymer concentration was increased the syringeability was reduced.

#### 3.3.4 pH

pH value of all *insitu* gel formulation ranged in between 5.8-6.4, indicating that the formulation was suitable and better for nasal administration with reduced irritation of tissue. pH of all the formulations is given in Table **2** and Fig. (**6b**).

#### 3.3.5 Spreadability Studies

The formulated *insitu* gel exhibited satisfactory spreadability indicating easy application of drug. The spreadability of the optimised *insitu* formulation was in the range of 34.33±1.42 g cm /second. Spreadability studies were shown in Fig. (**6c** and Table **2**.

#### 3.3.6 Gel Strength

Gel strength of different *insitu* gel formulation was in the range of 3.1±0.56 g and 7.7 ± 0.45 g as given in Fig. (**6d**) and Table **2**.

#### 3.3.7 Viscosity Studies

**Fig. (7):**
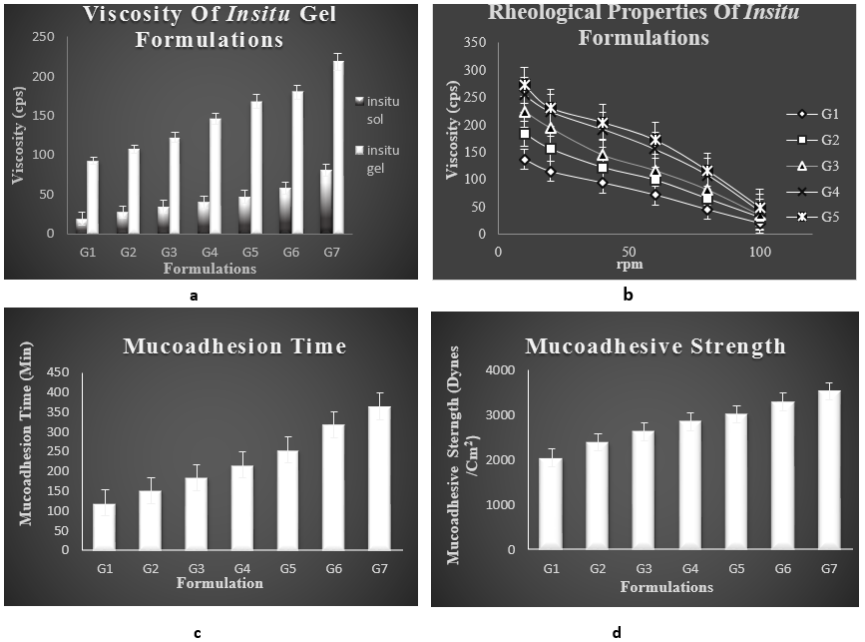
a) Viscosity; b)Rheological properties; c) Mucoadhesion time; d) Mucoadhesive strength of different formulations of *in situ* gel of lamotrigine.

One important pre-requisite for intranasal *in situ* gel was viscosity of the formulation. As indicated, a formulation suitable for application to the nasal cavity should ideally have a low viscosity when applied and after administration should have a high viscosity in order to stay at the application site. All the formulations of batches G1– G7 showed a polymer concentration dependent rise in viscosity. Viscosity of the both *insitu* sol and *insitu* gel was examined at 100rpm. G7 formulation was having maximum viscosity. The viscosity of G5 formulation was taken as optimum. Viscosity of *insitu* sol formulation ranged between 19± 0.27- 80.26± 0.53 cps. Viscosity of *insitu* gel ranged between 93± 0.52 to 218± 0.63 cps as shown in Fig. **7a** and Table (**2**).

#### 3.3.7 Rheological Studies

This is an important parameter for the *in situ* gels to be evaluated. This study excluded G6 and G7 formulations since these formulations do not have sufficient syringeability and such formulations are avoided because of difficulty in administration. The viscosity of formulations should be in an optimum range which improves its ease of administration. The flow curve (viscosity against speed / rpm) of selected formulations indicated that for the selected polymer concentrations, pseudoplastic systems with shear thinning property were obtained. The prepared formulations tend to thin when being exposed to shearing force. Fig. (**7b**) compares the shear dependent viscosity of prepared formulations containing gellan gum and xanthan gum.

#### 3.3.8 Drug content Estimation

The drug content was estimated for all prepared *in situ* gel formulation. Acceptable range of drug content in the range of 86.12± 0.32% - 97.6 ± 0.68% was present in all of them indicating uniform distribution of drug. The maximum amount of drug was present in G5 formulation with 97.6 ± 0.68%. The concentration of drug content proportionally depends on polymer concentration, But when polymer concentration is too high the concentration of drug was slightly less when compared to the former ones. The drug concentration was estimated to be maximum when 1.75%w/v of polymer was used. Drug content estimation was given in Table **3**.

### 3.4 *In vitro* Mucoadhesion Studies

#### 3.4.1 *In vitro* Mucoadhesion Time

Mucoadhesion time taken for the insitu gel to erode from the nasal mucosa was noted. The adhesion time taken for G5 formulation was found to be higher than other formulation. Mucoadhesion is imparted by the formulation due to the addition of a natural mucoadhesive polymer gellan gum. Bioadhesive time was found to be increasing with raising concentration of gellan gum. Higher mucoadhesive time of optimised formulation indicates that it can be attached to the nasal mucosa for longer period with less risk of mucocilliary clearance and provides a sustained effect. Mucoadhesive time of all the formulations is mentioned in Table **3** and Fig. (**7c**).

**Mucoadhesion can be explained by two stages:**

- The spreading and swelling of formulation when the mucoadhesive polymer binds to mucous membrane mark the first stage, *i.e* contact stage.
- The next stage is the activation of mucoadhesive polymer, which is due to the interaction of mucoadhesive polymer with the ions present on the nasal mucosa.

#### 3.4.2 *In vitro* Mucoadhesive Strength

Mucoadhesion studies were carried out to ensure that the formulation had the ability to adhere to the mucous membrane at the site of absorption for a long period of time. With increase in polymer concentration a raise in mucoadhesion was seen. The mucoadhesive character is exhibited by the polymer gellan gum, by interaction of the specific functional groups in polymer like hydroxyl and carboxyl group with the glycoprotein chain in the mucous layers of epithelial cells of nasal cavity. The interactions can be strong or weak like wander walls interaction, ionic bonding and hydrogen bonding. The polymer is an ion activated one which upon interaction with the physiological ions in the nasal electrolytes form gel. The interaction of the polymer with the glycoprotein chains in the mucin network occurs when the polymer swells by absorbing water from the mucous layer and adhesion binding occurs to form gel. The key factors that play a major role in mucoadhesion are the physiological ions in the nasal cavity as well as the swelling capacity of the polymer which finally result in prolonged mucoadhesion and reduced mucocilliary clearance. Mucoadhesive strength of all the formulations is mentioned in Fig. (**7d**) and Table **3**.

### 4.5 *In vitro* Release Studies

The *in vitro* release studies of different formulations of drug loaded *in situ* gels were carried out for 20minute in PBS pH6.6. PBS of pH6.6 was selected as medium for drug absorbance since it resembles nasal pH. Throughout the study the pH and temperature were kept constant. The maximum release was found to be for G5 and G4 with respective release of 97.02 ± 0.54% and 95.96 ±0.16%. The polymer concentration plays a key role in release pattern of drug. The release of drug mainly depends on the polymer concentration. Polymer concentration was proportional to release to some extent. But too high concentration of the polymer can adversely affect the *in vitro* release as in formulation G7. The G5 formulation was showing highest percentage release and was regarded as the optimised formulation. The drug: wax ratio was optimum for the G5 formulation which was the reason for its immediate release. Fig. (**8**) shows the *in vitro* release of different *in situ* gel formulations.

### 3.6 Kinetics

The G5 formulation was having the maximum drug release. Based on the *in vitro* drug release data kinetic study was done for optimised G5 formulation. The drug release mechanism was investigated by subjecting the release data of the optimised formulation to different kinetics models. On comparing the regression coefficient values of different kinetic models, it was observed that the R^2^ value of different kinetic models and release data were found to be 1^st^ order which was best fitted with Korsmeyer peppas model where the n value was greater than 0.5, indicating non fickan transport mechanism of drug release. Fig. (**9**) shows the different kinetic models of optimised *insitu* gel G5 formulation

### 3.7 *Ex vivo* Permeation Studies

**Fig. (10):**
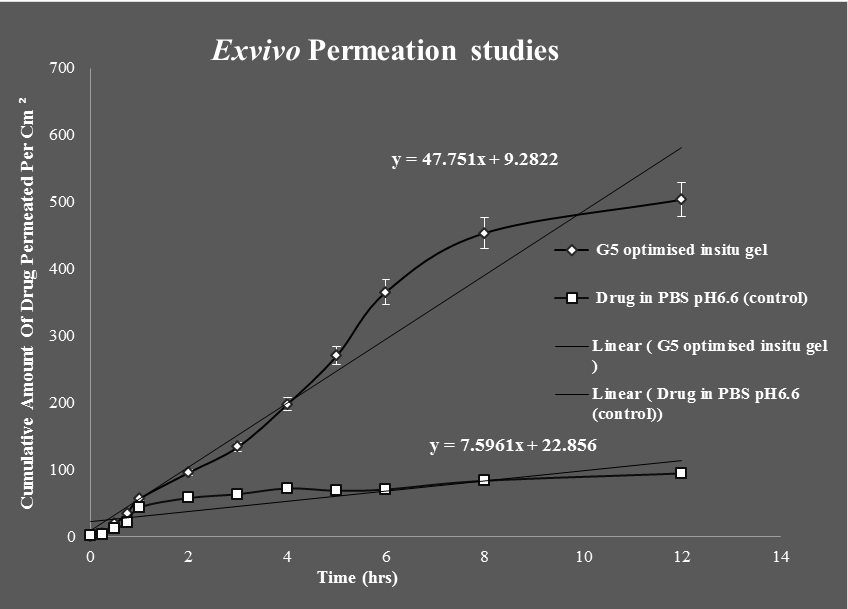
*Ex vivo* permeation studies of G5 *insitu* optimised gel and control formulations.

Franz diffusion cell was used for conducting *exvivo* permeation studies. The permeation studies were conducted by using bovine nasal mucosa for 12 hours. The bovine nasal mucosa was selected because of its physiological resemblance to human nasal mucosa. The nasal mucosa represents a layer with higher vascularisation. The *exvivo* permeation study was done for optimised G5 formulation and control formulation containing the drug in PBS pH6.6 and comparison studies done between them were plotted on a graph. G5 optimised formulation was showing maximum permeation than control indicating slow and sustained permeation of drug from nasal mucosa. The release process of the drug from the preparation depends on the dissolution from the gel in aqueous media and more viscous gels release lamotrigine more slowly than less viscous solutions. These results indicate that the viscosity of the *insitu* gel plays an important role in controlling the release of the drug if the dissolution of the drug through the polymer matrix is a rate determining step. The *exvivo* permeation studies of the above mentioned two formulations are given in (Fig. **10**).

Students T Test were carried out for statistical analysis of data and the P value was 0.0126 which was significant, since p value is < 0.05.Statistical analysis showed significant improvement in the *exvivo * permeation of the optimised G5* insitu* formulation than when compared to control.

*Ex vivo* studies revealed that amount of lamotrigine permeated per cm^2^ was improved significantly (P < 0.05) for G5 formulation than when compared to positive control.

The permeability coefficient and steady state flux values of the G5 *insitu* optimised formulation were higher than the positive control. Fig. (**11**) shows comparison of steady state flux between the optimised *insitu *G5 formulation and control containing drug in PBS at pH 6.6.

### 3.8 Histopathological Examination

Fig. (**12a** and **12b**) shows the histopathological study of nasal mucosa after permeation with optimised G5 formulation which does not cause any irritation or toxicity and was safe for nasal administration.

### 3.9 SEM

SEM studies Fig. (**13a**) show discrete, spherical shaped with a rough outer surface. Visible wrinkles are shown in the SEM studies.

### 3.10 TEM

The shape and morphology of the *insitu* gel formulation can be studied by TEM image. TEM image of the lamotrigine loaded *insitu* gel formulation is shown in Fig. (**13b**), indicating spherical particles in nanometre range which are homogeneously distributed throughout the formulation.

### 3.11 Stability Studies

Parameters like drug content and gel strength were evaluated from 0 day to 45 days at regular intervals in 3 different conditions *i.e*, refrigerated, room temperature and oven conditions. The optimised G5 *insitu* gel formulation was found to be more stable at refrigerated and room temperature conditions than in oven condition. Table **4** and Fig. (**14a**) a show the drug content and Table **4** and Fig. (**14b**) show gel strength of optimised *in situ* G5 formulation during stability studies over a period of 45 days.

## CONCLUSION

Epilepsy is a common chronic neurological disorder characterized by recurrent epileptic seizures. A number of delivery systems have been investigated but still a novel delivery system to combat this incurable firing disorder is yet to be developed. Intranasal drug delivery is one of the local drug delivery routes to combat this neurological disorder due to high vascularisation, larger surface area and porous endothelial membrane providing systemic effect and avoidance of first pass metabolism. It is also a non-invasive route. Antiepileptic drugs commonly used in treating epilepsy especially in conventional treatments are probably associated with many side effects. The main purpose of this study was to formulate an *insitu* gel drug delivery system with immediate release for treating epileptic condition that can minimize first pass metabolism and side effects by loading lamotrigine into different concentration of natural biodegradable polymeric solutions of gellan gum and xanthan gum that increases the mucoadhesion and residence time, reducing the mucocillary clearance and are also responsible for gelling property of the formulation. Among all the formulation G5 was fixed to be the optimised formulation, which had maximum drug release of 97.02±0.54% and greater permeation significantly (p < 0.05) than control. Histopathological studies showed there is no alteration in drug treated nasal mucosa after permeation with optimised formulation when compared with normal nasal mucosa, this indicates less irritation to the nasal mucosa. Administration of drug loaded *insitu* gel formulation into nasal cavity proves to be viable and effective alternative to other conventional therapy and in future its clinical use is promising.

## Figures and Tables

**Fig. (1):**
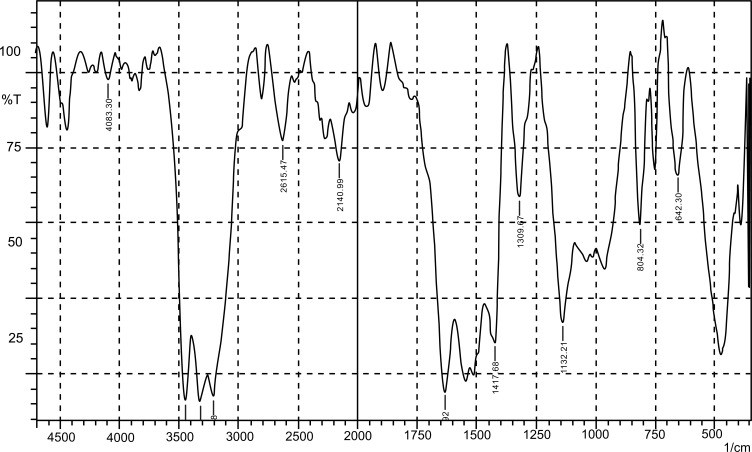
FTIR spectrum of sample lamotrigine.

**Fig. (4):**
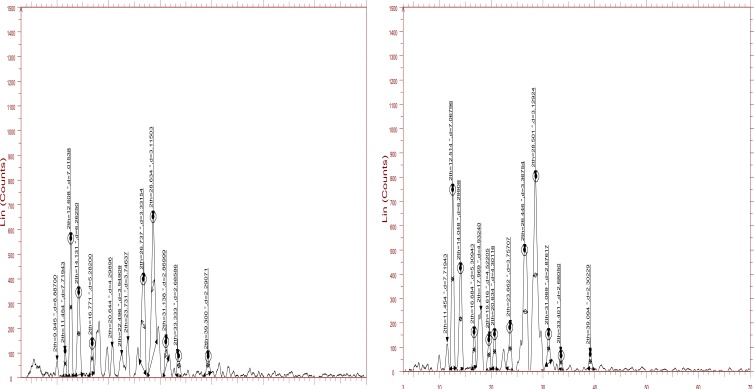
**a**) XRD pattern of *insitu* formulation; and b) XRD pattern of drug lamotrigine.

**Fig. (8):**
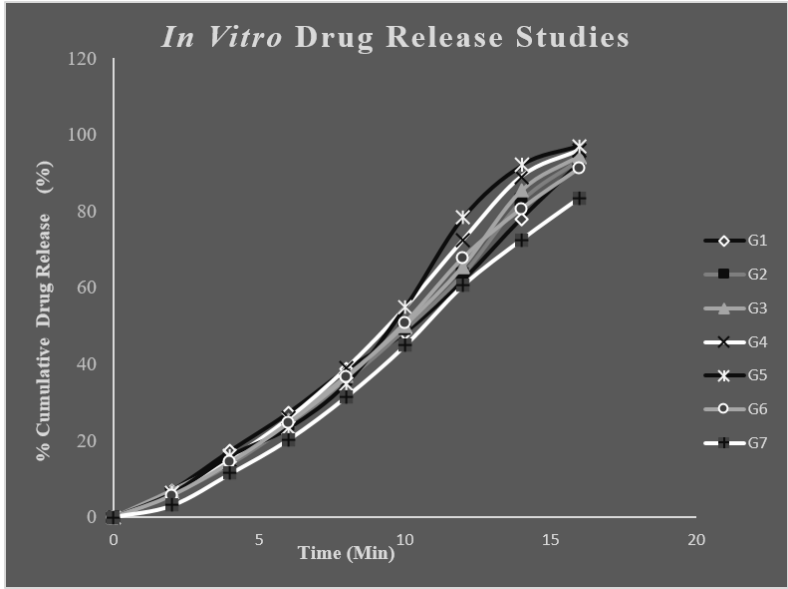
*In vitro* drug release studies of different *in situ* formulation.

**Fig. (9):**
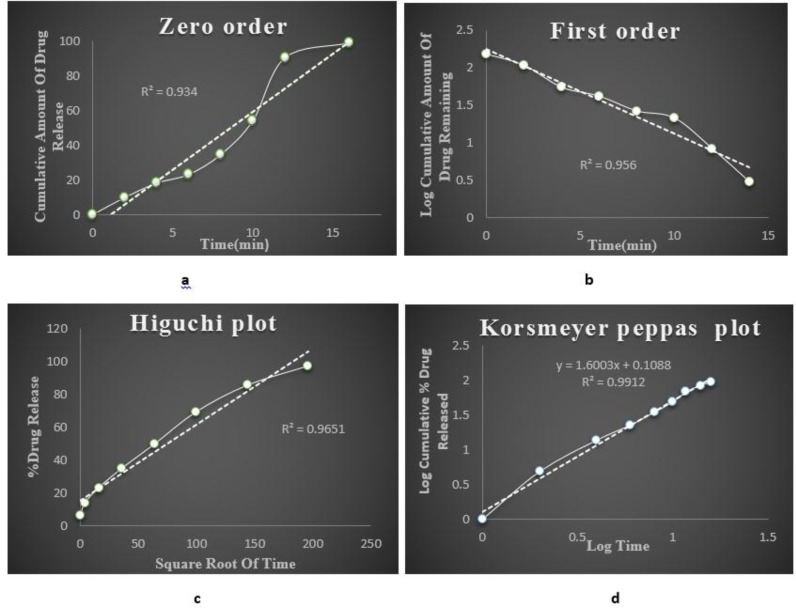
(a) Zero order kinetics; (b) first order kinetics; (c) Higuchi plot; (d) Korsmeyer peppas model.

**Fig. (11):**
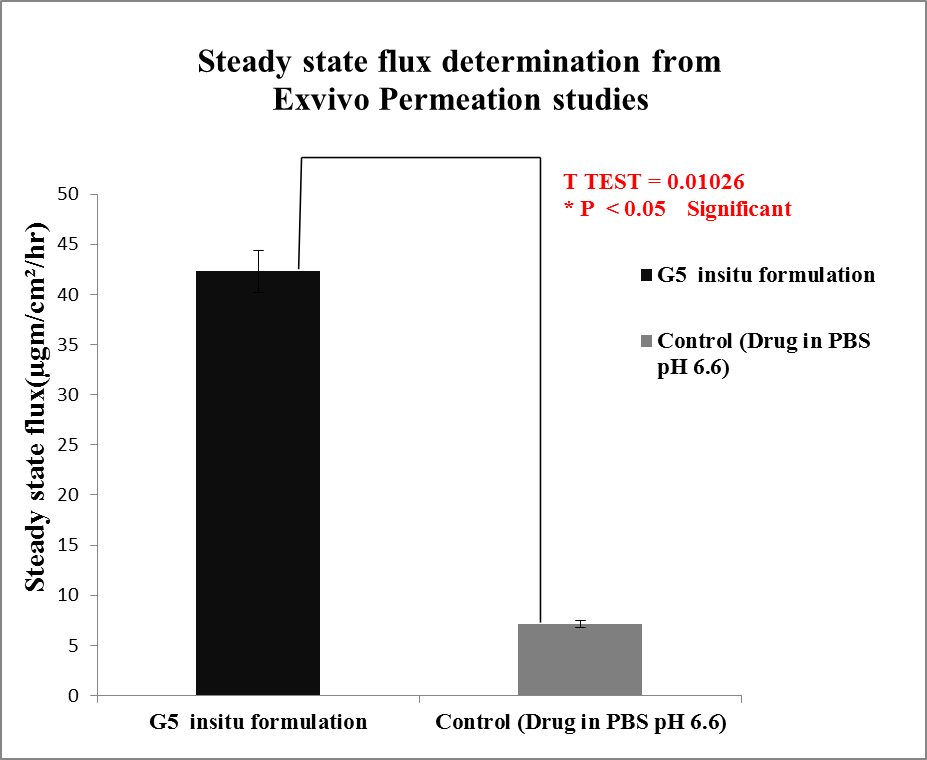
Steady state Flux determination from *exvivo* permeation studies of optimised G5 formulation and positive control.

**Fig. (12):**
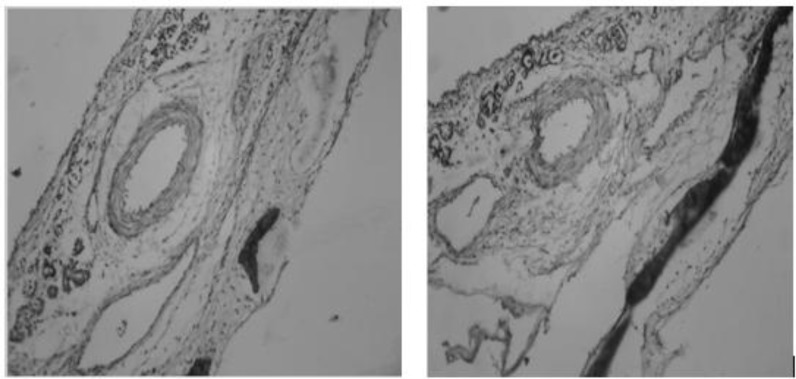
Histopathological evaluation of nasal mucosa. a) Normal mucosa; b) Mucosa treated with optimised formulation.

**Fig. (13):**
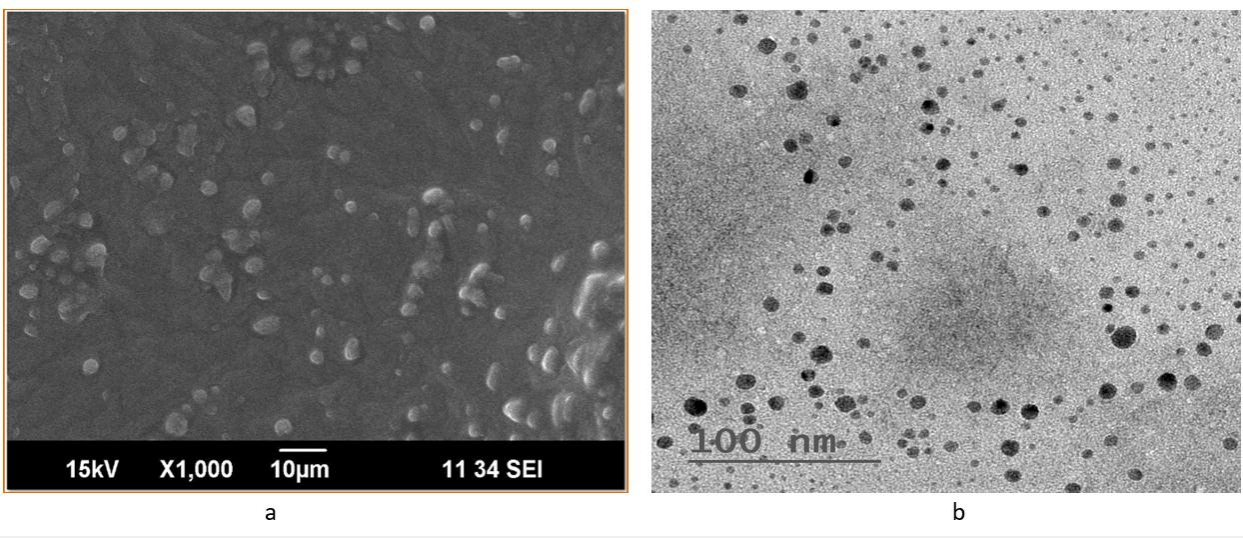
**a)** SEM image of lamotrigine loaded *insitu* gel formulation; **b)** TEM image of Lamotrigine loaded *insitu* gel formulation.

**Fig. (14):**
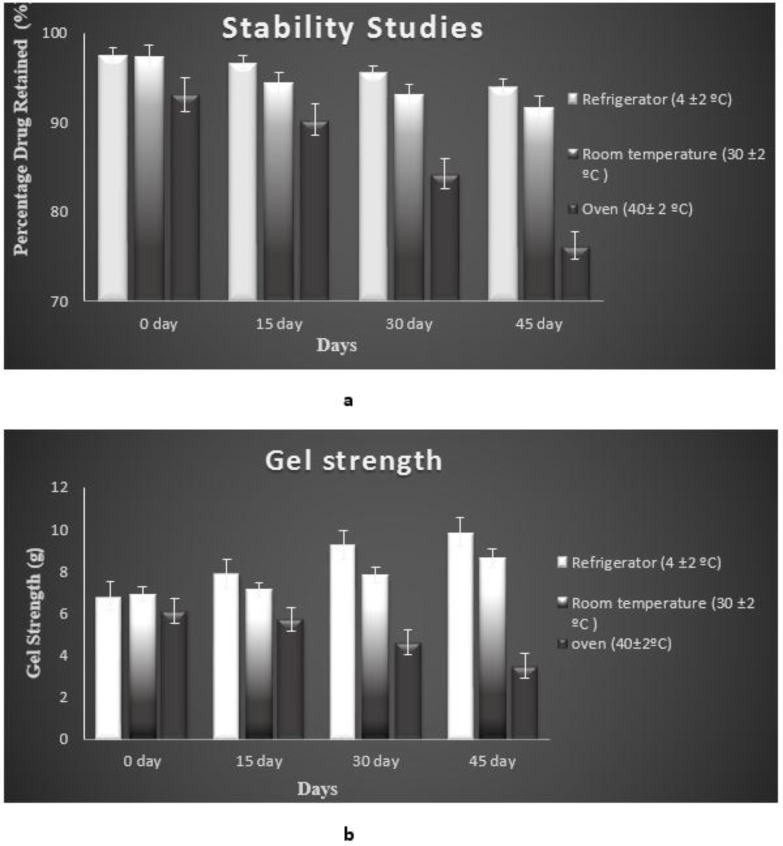
**a)** Drug content; **b**) Gel strength of optimised *insitu* gel G5 formulation during stability studies at different conditions.

**Table 1:** Composition of different formulation of intra nasal *insitu* gel of Lamotrigine.

SL.NO.	FORMULATION	LAMOTRIGINE (DRUG) %w/v	**GELLAN GUM %w/v**	XANTHAN GUM %w/v	MANNITOL %w/v	PEG%w/v	ETHYL PARABEN %w/v
1	**G_1_**	0.2	0.5	0.25	0.15	0.2	0.10
2	**G_2_**	0.2	0.75	0.25	0.15	0.2	0.10
3	**G_3_**	0.2	1.0	0.25	0.15	0.2	0.10
4	**G_4_**	0.2	1.25	0.25	0.15	0.2	0.10
5	**G_5_**	0.2	1.5	0.25	0.15	0.2	0.10
6	**G_6_**	0.2	1.75	0.25	0.15	0.2	0.10
7	**G_7_**	0.2	2.0	0.25	0.15	0.2	0.10

**Table 2:** Gelling time, Syringeability, pH, Spreadability, Gel strength, and Viscosity of different *insitu* gel formulations.

**Formulations**	**Gelling time (sec)**	**Syringeability**	**pH**	Spreadability (gcm/sec)*	**Gel Strength (g)**	**Viscosity (cps)**	
-	-	-	-	-	-	**sol**	**gel**
**G1**	11.3±0.21	Pass	5.8±0.01	15.24±0.72	3.1 ±0.56	19±0.27	93±0.52
**G2**	10.2±0.34	Pass	5.9±0.02	21.30±1.19	4.7 ±0.74	27±0.35	107.4±0.45
**G3**	9.5±0.41	Pass	6.1±0.02	25.15±1.24	5.9 ±0.81	34 ±0.25	122.2±0.11
**G4**	7.2±0.35	Pass	6.3±0.04	29.76±1.36	6.5 ±0.63	40.16±0.36	146.13±0.63
**G5**	5.2±0.61	Pass	6.5±0.02	34.33±1.42	6.9 ±0.94	46.83±0.33	168±0.85
**G6**	4.6±0.41	Fail	6.6±0.08	42.71±1.51	7.2 ±0.72	58.16±0.74	180±0.25
**G7**	3.4±0.52	Fail	6.8±0.05	56.19±1.66	7.7 ±1.25	80.26±0.53	218±0.63

(Values are expressed as mean ± standard deviation, n=3

**Table 3:** Drug content, Mucoadhesion time, and Mucoadhesive strength of different *in situ* gel formulations.

Formulations	Drug Content (%)	Mucoadhesive Time (min)	Mucoadhesive Strength (dynes/cm^2^)
**G1**	89.89 ±1.52	120min	2046 ±0.56
**G2**	92.16 ±0.34	151min	2393 ±0.42
**G3**	94.35 ±0.41	184min	2628 ±0.61
**G4**	96.10 ±0.85	216min	2853 ±0.75
**G5**	97.61 ±0.68	254min	3018 ±0.67
**G6**	93.75 ±0.96	318min	3295 ±0.72
**G7**	86.12 ±0.32	364min	3526 ±0.56

(Values are expressed as mean ± standard deviation, n=3)

**Table 4:** Stability studies of optimised G5 *insitu* gel formulation.

**Temperature**	**Drug Content**				**Gel Strength(g)**			
-	O day	15 day	30 day	45 day	0 day	15 day	30 day	45 day
**Refrigerator** **(4 ±2 ºC)**	97.2±0.48	97.7±0.62	96.6±0.64	95.1±0.51	6.8±0.64	7.9±0.91	9.25±1.94	9.86±2.41
**Room temperature(30±2ºC)**	97.9±0.23	97.4±0.56	96.1±0.52	94.3±0.46	6.9±0.41	7.1±0.94	7.8±1.34	8.6±1.64
**Oven 40± 2 ºC)**	96.1±0.33	90.3±0.35	84.3±0.51	76.2±0.54	6.1±0.53	5.7±1.25	4.6±2.19	3.5±2.45

(Values are expressed as mean ± standard deviation, n=3)
